# Overexpression of the long non-coding RNA PVT1 is correlated with leukemic cell proliferation in acute promyelocytic leukemia

**DOI:** 10.1186/s13045-015-0223-4

**Published:** 2015-11-06

**Authors:** Chengwu Zeng, Xibao Yu, Jing Lai, Lijiang Yang, Shaohua Chen, Yangqiu Li

**Affiliations:** First Affiliated Hospital, Jinan University, Guangzhou, 510632 China; Institute of Hematology, Medical College, Jinan University, Guangzhou, 510632 China; Key Laboratory for Regenerative Medicine of Ministry of Education, Jinan University, Guangzhou, 510632 China

**Keywords:** Long non-coding RNA, Acute promyelocytic leukemia, All-*trans* retinoic acid, Differentiation

## Abstract

**Background:**

Acute promyelocytic leukemia (APL) is associated with chromosomal translocation t(15;17), which results in the proliferation of morphologically abnormal promyelocytes. Gain of supernumerary copies of the 8q24 chromosomal region, which harbors MYC and PVT1, has been shown to be the most common secondary alteration in human APL. Increased MYC can accelerate the development of myeloid leukemia in APL. However, the role that the expression of the long non-coding RNA (lncRNA) PVT1 plays in the pathogenesis of APL remains largely unknown.

**Findings:**

In this study, we first analyzed the lncRNA PVT1 expression level in peripheral blood cells from 28 patients with de novo APL, and significantly upregulated PVT1 was found in APL patients compared with healthy donors. We then observed significantly lower MYC and PVT1 expression during all-*trans* retinoic acid (ATRA)-induced differentiation and cell cycle arrest in the APL cell line. MYC knockdown in NB4 cells led to PVT1 downregulation. Moreover, PVT1 knockdown by RNA interference led to suppression of the MYC protein level, and cell proliferation was inhibited.

**Conclusion:**

Our findings reveal that the lncRNA PVT1 may play an important role in the proliferation of APL cells and may be useful for future therapeutic management.

**Electronic supplementary material:**

The online version of this article (doi:10.1186/s13045-015-0223-4) contains supplementary material, which is available to authorized users.

## Background

Acute promyelocytic leukemia (APL) is characterized by a balanced reciprocal translocation between chromosomes 15 and 17, which leads to the expression of the fusion protein PML-RARα [1, 2]. All-*trans* retinoic acid (ATRA) and arsenic trioxide (ATO) have been used in APL therapy to induce the degradation of the key leukemogenic protein PML-RARα [3, 4]. Transcription factors such as PU.1 are involved in the pathogenesis of APL [3, 5, 6]. However, the precise mechanisms involved in APL pathogenesis beyond genetic alterations remain poorly understood [7].

Non-coding RNAs, such as microRNAs (miRNAs) and long non-coding RNAs (lncRNAs), have been implicated in the carcinogenesis of many different cancer types [8–11]. LncRNAs are non-protein coding transcripts longer than 200 nucleotides, and they account for a large proportion of the mammalian genome [12]. Recently, it has been suggested that lncRNAs are crucial for the development of malignant tumors [13–15]. LncRNAs have been demonstrated to regulate gene expression through epigenetic, transcriptional, and posttranscriptional regulation, and they are involved in X chromosome silencing, genomic imprinting, chromatin modifications, and other important regulatory processes [14, 16–21]. For example, a recent study has indicated that HOTAIRM1 (HOX transcript antisense RNA) provides a regulatory link in myeloid maturation by modulating integrin-controlled cell cycle progression at the gene expression level [22]. We previously demonstrated that lncRNAs play a significant role in regulating differentiation in APL cells [13].

PML-RARα is an initiating factor for APL leukemogenesis [23–26]. However, leukemia development in transgenic mice expressing PML-RARα occurs after a long latency period [27], which strongly suggests that PML-RARα collaborates with additional genetic lesions to block differentiation and promote leukemia. In fact, gain of supernumerary copies of the 8q24 chromosomal region has been shown to be the most common secondary alteration in human APL [28]. The lncRNA PVT1 is located on chromosome 8q24, a location shared with the well-known oncogene *c-myc* [29, 30]. Chromosome 8q24 has an equivalent in mice (chromosome 15), which is the most commonly recurring abnormality in PML-RARα transgenic mice [28], and it cooperates with PML-RARα to accelerate the development of myeloid leukemia [31]. Previous studies have focused on the *c-myc* oncogene, and it remains unknown whether the lncRNA PVT1 in the same region is also involved in leukemia. In this study, we aimed to characterize the role and regulation of PVT1 in APL.

## Results

### PVT1 is upregulated in APL

**Fig. 1:**
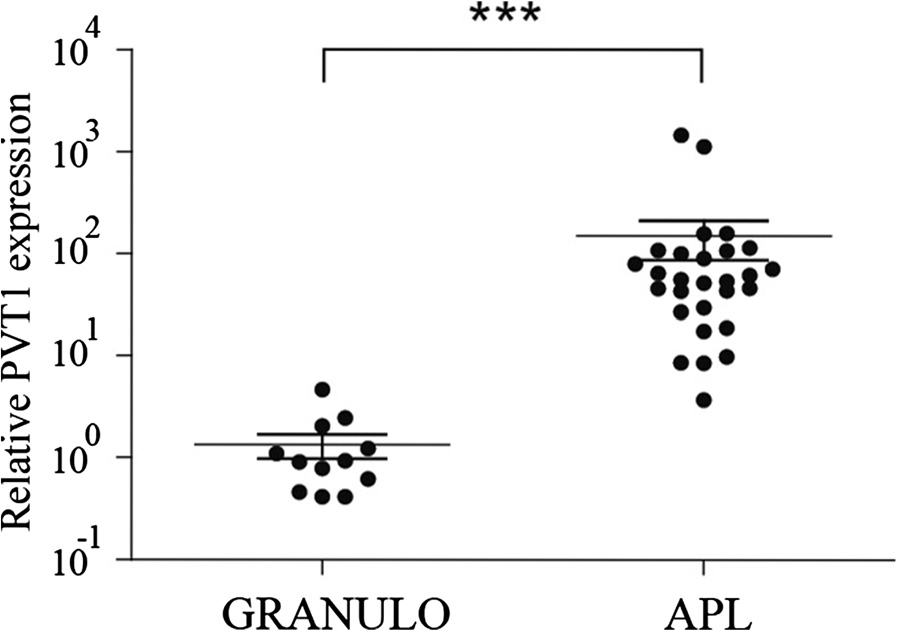
The lncRNA PVT1 is significantly upregulated in APL patient samples. Comparison of PVT1 expression in granulocytes from healthy donors (normal, *n* = 12) compared with APL cells (*n* = 28). PVT1 expression was detected by qRT-PCR and normalized to the ACTB gene. The expression of PVT1 relative to that in healthy samples was calculated using the 2^−DeltaDeltaCt^ method. *P* values between samples were obtained by performing a *t* test. *** p < 0.001

To investigate whether PVT1 is involved in the development of APL, we initially compared the PVT1 expression in primary APL patient samples with that in healthy donors. As shown in Fig. 1, the PVT1 expression level was significantly elevated in APL samples compared with healthy donors. This result suggests that PVT1 upregulation may be associated with the pathogenesis of APL cells.

### ATRA treatment represses PVT1 expression

**Fig. 2:**
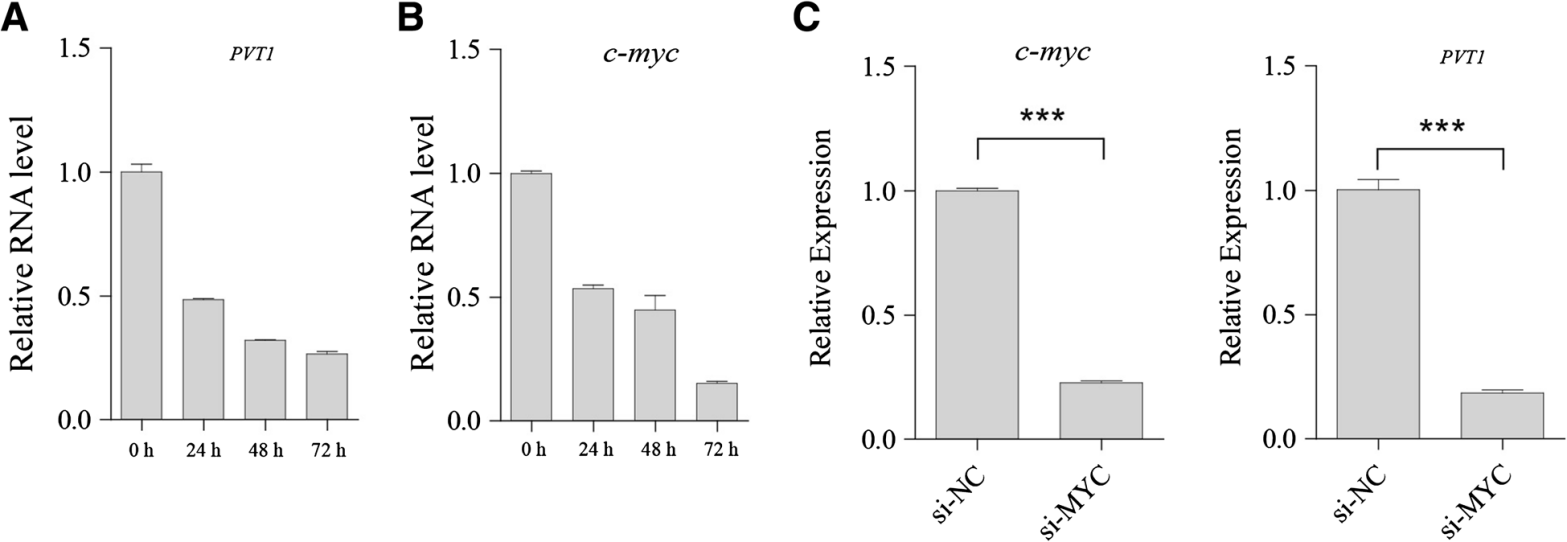
PVT1 was significantly decreased in NB4 cells treated with ATRA. **a** NB4 cells were treated with 1 μM ATRA. PVT1 was measured by qRT-PCR and normalized to the house keeping gene ACTB. **b** The level of *c-myc* mRNA in NB4 cells treated with ATRA was detected by qPCR. ATRA treatment demonstrated broadly similar effects on MYC and PVT1 expression. Each panel shows the mean ± SD of a representative experiment performed in triplicate. **c** qRT-PCR analysis of MYC and PVT1 in APL cells after MYC knockdown

Because ATRA treatment leads to a proliferation block and the differentiation of leukemia blasts, we next investigated PVT1 expression in the APL cell line NB4 before and after ATRA treatment (1 μM). Using qRT-PCR, the PVT1 expression level in NB4 cells was downregulated upon treatment with ATRA (Fig. 2a). There was no PVT1 downregulation in an ATRA-resistant cell line (NB4-R2) upon ATRA treatment, excluding a non-specific stress response to ATRA treatment (Additional file 1: Figure S1). Because it was reported that the well-known protein MYC is a PVT1 transcriptional activator, we further investigated the effects of ATRA on *c-myc* expression. Consistent with previous studies [32], treatment of APL cells with ATRA inhibited the expression of *c-myc* messenger RNA (mRNA) (Fig. 2b). Because of the remarkable association between PVT1 expression and MYC revealed by this study and others, we further investigated the effects of MYC on PVT1 expression. As shown in Fig. 2c, knockdown of MYC in NB4 cells led to PVT1 downregulation. These data suggest that PVT1 may be regulated by MYC and is involved in the proliferation of APL cells.

### Knockdown of PVT1 impairs the proliferation of APL cells

**Fig. 3:**
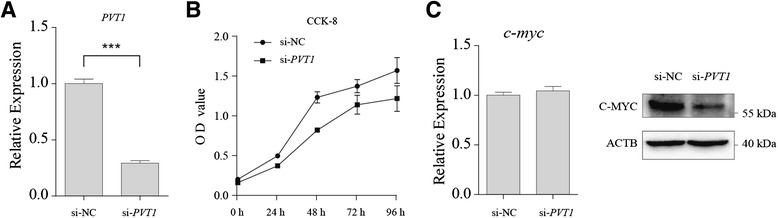
PVT1 inhibition attenuates the proliferation of APL cells. The PVT1 expression level was detected in NB4 cells transfected with siRNA specifically targeting PVT1 (si-*PVT1*) or negative control siRNA (si-NC). **a** qRT-PCR analysis showed that the PVT1 expression level in cells transfected with si-*PVT1* was significantly lower than that in cells transfected with si-NC. **b** PVT1 knockdown impaired the growth of APL Cells. The data are the result of three independent experiments and are presented as means ± SD. **c** qRT-PCR and Western blot analysis of MYC in APL cells after siRNA transfection

Based on the above data, we further elucidated the function of PVT1 during proliferation using CCK-8 assays. Reduced PVT1 expression in cells transfected with PVT1-specific small interfering RNA (siRNA) was confirmed by qRT-PCR (Fig. 3a). As shown in Fig. 3b, cells transfected *si-PVT1* had a lower survival rate than those in the control group. In an effort to determine whether PVT1 is involved in regulating the oncoprotein MYC, we examined the MYC expression level in cells transfected si-*PVT1*. PVT1 knockdown had no effect on *c-myc* RNA but led to the suppression of the MYC protein level in NB4 cells (Fig. 3c). These results suggest that the PVT1 lncRNA is involved in abnormal APL cell proliferation.

## Discussion

Leukemia is a hematologic disease in which cells are blocked at a certain stage of hematopoietic differentiation and display a high proliferative capacity [7, 33]. Recently, increasing evidence has suggested that lncRNAs are involved in fundamental biological processes, such as cell proliferation, survival, and differentiation [14, 18]. In this study, we reveal for the first time that the lncRNA PVT1 is significantly upregulated in primary APL cells. Additionally, we provide evidence that upregulated PVT1 expression is involved in the proliferation of APL cells.

More recently, the lncRNA PVT1 has been shown to be dysregulated in several cancers, and it has been functionally linked to cancer tumorigenesis [34–37]. PVT1 is an lncRNA (1.9 kb) and host gene for several miRNAs [38]. Although there are a few reports demonstrating that PVT1 plays an important role in the pathogenesis of several cancers, it is not yet clear whether PVT1 is involved in the regulation of APL, which is a unique subtype of acute myeloid leukemia (AML) that results from a blockade in granulocyte differentiation during the promyelocytic stage. Here, we found that PVT1 expression is elevated in APL, and its expression is repressed during ATRA-induced differentiation and cell cycle arrest. *c-myc* and PVT1 were located on chromosome 8q24; gain of supernumerary copies of the 8q24 chromosomal region in human APL may led to increased copy number of PVT1 in APL. In addition to a gain in 8q24, the well-known MYC protein is a transcriptional activator of PVT1 [32] and increased in human APL [31], and it has been reported that treating APL cells with ATRA inhibits the expression of *c-myc* mRNA [39], suggesting that elevated PVT1 expression may also result from MYC protein activation in APL cells. Similarly, in our study, *c-myc* knockdown led to PVT1 downregulation. Interestingly, PVT1 inhibition could attenuate the proliferation of APL cells, indicating that PVT1 is essential for APL progression. A recent study has found that PVT1 increases NOP2 levels by enhancing the stability of the NOP2 protein in hepatocellular carcinoma and that the function of PVT1 in cell proliferation is dependent on the presence of NOP2 [34]. More importantly, a recent report has also highlighted the involvement of PVT1 in regulating MYC, which has been firmly established to play a role in cancer [40]. Indeed, we confirmed that PVT1 depletion causes a reduction in the MYC protein level in APL. Thus, PVT1 may influence the stability of these important proteins, which are indispensable for APL cell growth. It is therefore possible that PVT1 could be a novel biomarker for APL diagnosis, prognosis, and targeted therapy [41].

In conclusion, we demonstrated that abnormal elevated expression of the lncRNA PVT1 may be correlated with APL cell proliferation. This is, to our knowledge, the first report of a potential role for the lncRNA PVT1 in APL. These findings provide new insight into the mechanism of APL progression.

## Materials and methods

### Samples

A total of 40 peripheral blood samples including 28 APL samples taken from the time of diagnosis and 12 samples from healthy donors were included in this study. The patient characteristics are summarized in Table 1. The peripheral blood mononuclear cells (PBMCs) of APL samples were isolated using Ficoll–Hypaque gradient centrifugation method [42, 43]. Granulocytes from healthy donors were isolated as previous studies [44]. All of the procedures were conducted according to the guidelines of the Medical Ethics Committee of the Health Bureau of the Guangdong Province of China. This study was approved by the Ethics Committee of the Medical School of Jinan University.

**Table 1:** APL patient characteristics

APL primary (*N* = 28)	Characteristics	Median (range)	No. (%)
	Cytogenetics		
	t(15;17)		28 (100)
	Age at diagnosis, year	26.58	26 (92.9)
	N/A		2 (7.1)
	Sex		
	Male		12 (42.9)
	Female		16 (57.1)
	WBC count (×10^9^/L)	22.93	
	Less than 10		10 (35.7)
	10–50		9 (32.1)
	50 or higher		2 (7.1)
	N/A		7 (25)
	Percent PB blasts	64.1	
	Below 80 %		10 (35.8)
	80 % or above		9 (32.1)
	N/A		9 (32.1)

*WBC* white blood cells, *PB* peripheral blood, *N/A* not available

### Cell lines and cell cultures

The NB4 and NB4-R2 cell lines were kindly provided by Dr. Yueqin Chen (Sun Yat-sen University, Guangzhou, China) and cultured in RPMI 1640 (HyClone, SH30027) containing 10 % fetal bovine serum (HyClone, SV30160) at 37 °C in a 5 % CO_2_ incubator. ATRA was purchased from Sigma-Aldrich and used at a final concentration of 1 μM (Sigma-Aldrich, R2625; stock: 10 mM in EtOH).

### RNA extraction and qRT-PCR analysis

Total RNA was extracted with TRIzol reagent (Invitrogen) according to the manufacturer’s protocol. RNA was reverse transcribed into cDNA using a Reverse Transcription Kit (Takara, Japan). Real-time PCR was performed with SYBR Green (TIANGEN, China). ACTB was used as a reference for both mRNA and lncRNA, and each sample was analyzed in triplicate. The primers used are as follows: PVT1: 5′-TGAGAACTGTCCTTACGTGACC-3′ (sense) and 5′-AGAGCACCAAGACTGGCTCT-3′ (antisense). ACTB: 5′-TTGTTACAGGAAGTCCCTTGCC-3′ (sense) and 5′-ATGCTATCACCTCCCCTGTGTG-3′ (antisense). *c-myc*: 5′-GGACGACGAGACCTTCATCAA-3′ (sense) and 5′-CCAGCTTCTCTCAGACGAGCTT-3′ (antisense) [45].

### RNA interference

*c-myc* siRNA (CGAUGUUGUUUCUGUGGAA) [46], *PVT1* siRNA (PVT-1 siRNA-1: GCUUGGAGGCUGAGGAGUUTT and *PVT-1* siRNA-2: CCCAACAGGAGGACAGCUUTT) [47], and negative control siRNA (siN05815122147) were purchased from RiboBio (Guangzhou, China). The siRNA oligonucleotides were transfected into NB4 cells using the Neon® Transfection System (Invitrogen) following the manufacturer’s protocol [48].

### Cell proliferation assays

Cell proliferation was quantified daily on days 0–4 with the CCK-8 kit (Dojindo, Japan) according to the manufacturer’s protocol. NB4 cells were plated at a density of 1 × 10^4^ cells/well in 96-well plates and cultured in RPMI 1640 medium containing 10 % FBS. The CCK-8 reagent (10 μL) was added to the wells at the end of the experiment. After incubation at 37 °C for 4 h, the absorbance in each well was determined using a microplate reader at 450 nm.

## Additional file

Additional file 1: Figure S1. PVT1 expression in NB4-R2 cells treated with ATRA. NB4-R2 cells were cultured in RPMI 1640 supplemented with 10 % FBS in the presence and absence of 1 μM ATRA for the indicated time points. Total RNA was extracted, and PVT1 levels were determined by qPCR. (DOCX 51 kb)

## Abbreviations

APL: acute promyelocytic leukemia
ATRA: all-*trans* retinoic acid
lncRNA: long non-coding RNA
qRT-PCR: quantitative reverse transcription-PCR
siRNA: small interfering RNA

## References

[1] Grignani F, Ferrucci PF, Testa U, Talamo G, Fagioli M, Alcalay M (1993). The acute promyelocytic leukemia-specific PML-RAR alpha fusion protein inhibits differentiation and promotes survival of myeloid precursor cells. Cell.

[2] Duprez E, Wagner K, Koch H, Tenen DG (2003). C/EBPbeta: a major PML-RARA-responsive gene in retinoic acid-induced differentiation of APL cells. EMBO J.

[3] Wang K, Wang P, Shi J, Zhu X, He M, Jia X (2010). PML/RARalpha targets promoter regions containing PU.1 consensus and RARE half sites in acute promyelocytic leukemia. Cancer Cell.

[4] Zeng CW, Chen ZH, Zhang XJ, Han BW, Lin KY, Li XJ (2014). MIR125B1 represses the degradation of the PML-RARA oncoprotein by an autophagy-lysosomal pathway in acute promyelocytic leukemia. Autophagy.

[5] Qian M, Jin W, Zhu X, Jia X, Yang X, Du Y (2013). Structurally differentiated cis-elements that interact with PU.1 are functionally distinguishable in acute promyelocytic leukemia. J Hematol Oncol.

[6] Mueller BU, Pabst T, Fos J, Petkovic V, Fey MF, Asou N (2006). ATRA resolves the differentiation block in t(15;17) acute myeloid leukemia by restoring PU.1 expression. Blood.

[7] Ablain J, de The H (2011). Revisiting the differentiation paradigm in acute promyelocytic leukemia. Blood.

[8] Loewen G, Jayawickramarajah J, Zhuo Y, Shan B (2014). Functions of lncRNA HOTAIR in lung cancer. J Hematol Oncol.

[9] Dzikiewicz-Krawczyk A (2014). MicroRNA-binding site polymorphisms in hematological malignancies. J Hematol Oncol.

[10] Wang WT, Chen YQ (2014). Circulating miRNAs in cancer: from detection to therapy. J Hematol Oncol.

[11] Zeng CW, Zhang XJ, Lin KY, Ye H, Feng SY, Zhang H (2012). Camptothecin induces apoptosis in cancer cells via microRNA-125b-mediated mitochondrial pathways. Mol Pharmacol.

[12] Kapranov P, Cheng J, Dike S, Nix DA, Duttagupta R, Willingham AT (2007). RNA maps reveal new RNA classes and a possible function for pervasive transcription. Science.

[13] Zeng C, Xu Y, Xu L, Yu X, Cheng J, Yang L (2014). Inhibition of long non-coding RNA NEAT1 impairs myeloid differentiation in acute promyelocytic leukemia cells. BMC Cancer.

[14] Morlando M, Ballarino M, Fatica A (2015). Long non-coding RNAs: new players in hematopoiesis and leukemia. Front Med.

[15] Xu TP, Huang MD, Xia R, Liu XX, Sun M, Yin L (2014). Decreased expression of the long non-coding RNA FENDRR is associated with poor prognosis in gastric cancer and FENDRR regulates gastric cancer cell metastasis by affecting fibronectin1 expression. J Hematol Oncol.

[16] Zhang H, Chen Z, Wang X, Huang Z, He Z, Chen Y. Long non-coding RNA: a new player in cancer. J Hematol Oncol. 2013;6:37. 10.1186/1756-8722-6-37 PMC3693878 23725405

[17] Zhuang Y, Wang X, Nguyen HT, Zhuo Y, Cui X, Fewell C (2013). Induction of long intergenic non-coding RNA HOTAIR in lung cancer cells by type I collagen. J Hematol Oncol.

[18] Garzon R, Volinia S, Papaioannou D, Nicolet D, Kohlschmidt J, Yan PS (2014). Expression and prognostic impact of lncRNAs in acute myeloid leukemia. Proc Natl Acad Sci U S A.

[19] Guo G, Kang Q, Chen Q, Chen Z, Wang J, Tan L (2014). High expression of long non-coding RNA H19 is required for efficient tumorigenesis induced by Bcr-Abl oncogene. FEBS Lett.

[20] Imamura K, Imamachi N, Akizuki G, Kumakura M, Kawaguchi A, Nagata K (2014). Long noncoding RNA NEAT1-dependent SFPQ relocation from promoter region to paraspeckle mediates IL8 expression upon immune stimuli. Mol Cell.

[21] Kam Y, Rubinstein A, Naik S, Djavsarov I, Halle D, Ariel I (2014). Detection of a long non-coding RNA (CCAT1) in living cells and human adenocarcinoma of colon tissues using FIT-PNA molecular beacons. Cancer Lett.

[22] Zhang X, Weissman SM, Newburger PE (2014). Long intergenic non-coding RNA HOTAIRM1 regulates cell cycle progression during myeloid maturation in NB4 human promyelocytic leukemia cells. RNA Biol.

[23] Nasr R, Guillemin MC, Ferhi O, Soilihi H, Peres L, Berthier C (2008). Eradication of acute promyelocytic leukemia-initiating cells through PML-RARA degradation. Nat Med.

[24] Lallemand-Breitenbach V, Zhu J, Chen Z, de The H (2012). Curing APL through PML/RARA degradation by As2O3. Trends Mol Med.

[25] Kogan SC (2009). Curing APL: differentiation or destruction?. Cancer Cell.

[26] Ward SV, Sternsdorf T, Woods NB (2011). Targeting expression of the leukemogenic PML-RARalpha fusion protein by lentiviral vector-mediated small interfering RNA results in leukemic cell differentiation and apoptosis. Hum Gene Ther.

[27] Walter MJ, Park JS, Lau SK, Li X, Lane AA, Nagarajan R (2004). Expression profiling of murine acute promyelocytic leukemia cells reveals multiple model-dependent progression signatures. Mol Cell Biol.

[28] Le Beau MM, Bitts S, Davis EM, Kogan SC (2002). Recurring chromosomal abnormalities in leukemia in PML-RARA transgenic mice parallel human acute promyelocytic leukemia. Blood.

[29] Shtivelman E, Bishop JM (1990). Effects of translocations on transcription from PVT. Mol Cell Biol.

[30] Chinen Y, Sakamoto N, Nagoshi H, Taki T, Maegawa S, Tatekawa S (2014). 8q24 amplified segments involve novel fusion genes between NSMCE2 and long noncoding RNAs in acute myelogenous leukemia. J Hematol Oncol.

[31] Jones L, Wei G, Sevcikova S, Phan V, Jain S, Shieh A (2010). Gain of MYC underlies recurrent trisomy of the MYC chromosome in acute promyelocytic leukemia. J Exp Med.

[32] Carramusa L, Contino F, Ferro A, Minafra L, Perconti G, Giallongo A (2007). The PVT-1 oncogene is a Myc protein target that is overexpressed in transformed cells. J Cell Physiol.

[33] Theilgaard-Monch K, Jacobsen LC, Borup R, Rasmussen T, Bjerregaard MD, Nielsen FC (2005). The transcriptional program of terminal granulocytic differentiation. Blood.

[34] Wang F, Yuan JH, Wang SB, Yang F, Yuan SX, Ye C (2014). Oncofetal long noncoding RNA PVT1 promotes proliferation and stem cell-like property of hepatocellular carcinoma cells by stabilizing NOP2. Hepatology.

[35] Yang YR, Zang SZ, Zhong CL, Li YX, Zhao SS, Feng XJ (2014). Increased expression of the lncRNA PVT1 promotes tumorigenesis in non-small cell lung cancer. I J Clin Exp Pathol.

[36] Huang C, Yu W, Wang Q, Cui H, Wang Y, Zhang L (2015). Increased expression of the lncRNA PVT1 is associated with poor prognosis in pancreatic cancer patients. Minerva Med.

[37] Ding J, Li D, Gong M, Wang J, Huang X, Wu T (2014). Expression and clinical significance of the long non-coding RNA PVT1 in human gastric cancer. OncoTargets Ther.

[38] Huppi K, Volfovsky N, Runfola T, Jones TL, Mackiewicz M, Martin SE (2008). The identification of microRNAs in a genomically unstable region of human chromosome 8q24. Mol Cancer Res.

[39] Dimberg A, Bahram F, Karlberg I, Larsson LG, Nilsson K, Oberg F (2002). Retinoic acid-induced cell cycle arrest of human myeloid cell lines is associated with sequential down-regulation of c-Myc and cyclin E and posttranscriptional up-regulation of p27(Kip1). Blood.

[40] Tseng YY, Moriarity BS, Gong W, Akiyama R, Tiwari A, Kawakami H (2014). PVT1 dependence in cancer with MYC copy-number increase. Nature.

[41] Smith AD, Roda D, Yap TA (2014). Strategies for modern biomarker and drug development in oncology. J Hematol Oncol.

[42] Gimenes-Teixeira HL, Lucena-Araujo AR, Dos Santos GA, Zanette DL, Scheucher PS, Oliveira LC (2013). Increased expression of miR-221 is associated with shorter overall survival in T-cell acute lymphoid leukemia. Exp Hematol Oncol.

[43] Xu L, Xu Y, Jing Z, Wang X, Zha X, Zeng C (2014). Altered expression pattern of miR-29a, miR-29b and the target genes in myeloid leukemia. Exp Hematol Oncol.

[44] Kusumanto YH, Dam WA, Hospers GA, Meijer C, Mulder NH (2003). Platelets and granulocytes, in particular the neutrophils, form important compartments for circulating vascular endothelial growth factor. Angiogenesis.

[45] Wall M, Poortinga G, Hannan KM, Pearson RB, Hannan RD, McArthur GA (2008). Translational control of c-MYC by rapamycin promotes terminal myeloid differentiation. Blood.

[46] Huang HL, Weng HY, Wang LQ, Yu CH, Huang QJ, Zhao PP (2012). Triggering Fbw7-mediated proteasomal degradation of c-Myc by oridonin induces cell growth inhibition and apoptosis. Mol Cancer Ther.

[47] Takahashi Y, Sawada G, Kurashige J, Uchi R, Matsumura T, Ueo H (2014). Amplification of PVT-1 is involved in poor prognosis via apoptosis inhibition in colorectal cancers. Br J Cancer.

[48] Miyake M, Hayashi S, Iwasaki S, Chao G, Takahashi H, Watanabe K (2010). Possible role of TIEG1 as a feedback regulator of myostatin and TGF-beta in myoblasts. Biochem Biophys Res Commun.

