# Supplementary material for: Overexpression of the long non-coding RNA PVT1 is correlated with leukemic cell proliferation in acute promyelocytic leukemia
Source: J Hematol Oncol. 2015 Nov 6;8:126. doi: 10.1186/s13045-015-0223-4 (PMC4636781; doi:10.1186/s13045-015-0223-4)

**Additional file 1: Figure S1.** PVT1 expression in NB4-R2 cells treated with ATRA. NB4-R2 cells were cultured in RPMI 1640 supplemented with 10% FBS in the presence and absence of 1 μM ATRA for the indicated time points. Total RNA was extracted, and PVT1 levels were determined by qPCR.


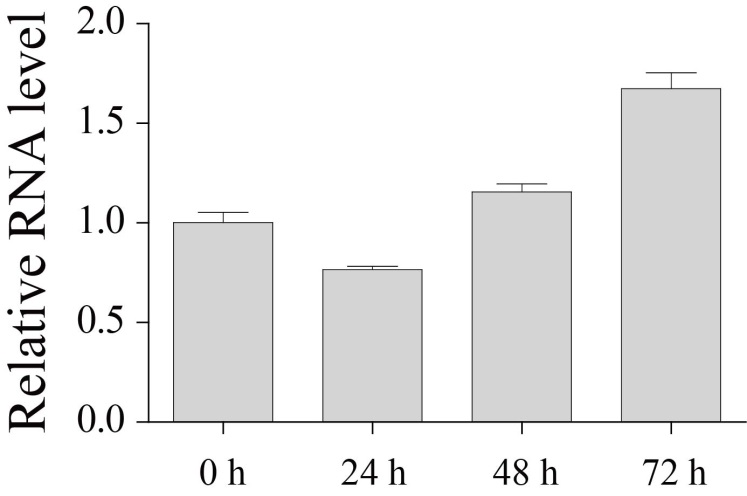

Supplement: Additional file 1: Figure S1. — PVT1 expression in NB4-R2 cells treated with ATRA. NB4-R2 cells were cultured in RPMI 1640 supplemented with 10 % FBS in the presence and absence of 1 μM ATRA for the indicated time points. Total RNA was extracted, and PVT1 levels were determined by qPCR. (DOCX 51 kb) [file 13045_2015_223_MOESM1_ESM.docx]
